# Regenerative medicine: current research and perspective in pediatric surgery

**DOI:** 10.1007/s00383-023-05438-6

**Published:** 2023-04-04

**Authors:** Koichi Deguchi, Elisa Zambaiti, Paolo De Coppi

**Affiliations:** 1 Stem Cells and Regenerative Medicine Section, University College London Great Ormond Street Institute of Child Health, London, UK; 2 NIHR BRC SNAPS Great Ormond Street Hospitals, London, UK; 3 Stem Cells and Regenerative Medicine Section, Faculty of Population Health Sciences, UCL Great Ormond Street Institute of Child Health, 30 Guilford Street, London, WC1N 1EH UK; 4 Present Address: Department of Pediatric Surgery, Osaka University Graduate School of Medicine, Osaka, Japan; 5 Present Address: UOC Chirurgia Pediatrica, Ospedale Infantile Regina Margherita, Turin, Italy

**Keywords:** Stem cell, ES cell, iPS cell, Regenerative medicine, Paediatric surgery, Tissue engineering, Organoid

## Abstract

The field of regenerative medicine, encompassing several disciplines including stem cell biology and tissue engineering, continues to advance with the accumulating research on cell manipulation technologies, gene therapy and new materials. Recent progress in preclinical and clinical studies may transcend the boundaries of regenerative medicine from laboratory research towards clinical reality. However, for the ultimate goal to construct bioengineered transplantable organs, a number of issues still need to be addressed. In particular, engineering of elaborate tissues and organs requires a fine combination of different relevant aspects; not only the repopulation of multiple cell phenotypes in an appropriate distribution but also the adjustment of the host environmental factors such as vascularisation, innervation and immunomodulation. The aim of this review article is to provide an overview of the recent discoveries and development in stem cells and tissue engineering, which are inseparably interconnected. The current status of research on tissue stem cells and bioengineering, and the possibilities for application in specific organs relevant to paediatric surgery have been specifically focused and outlined.

## Introduction

Regenerative medicine encompasses numerous areas of biology and engineering, including stem cell biology, biomaterial engineering and gene therapy [1]. The ultimate aim is to eventually creating functional “artificial” organs and tissues. Its enormous potential to engineer biologic organ substitutes, which can replace damaged organ and tissues of the patients, may fulfil the unmet clinical needs and overcome some of the unacceptable consequences of current therapeutic approaches in paediatric surgery, especially in the severe forms of congenital malformations and end-stage organ failures. Inspired by initial success in both preclinical and clinical studies using relatively simple organ systems and techniques, the research field has further expanded in an attempt to create more complex organs with sophisticated technologies.

This review will first briefly overview technological platforms available for regenerative medicine both after birth and prenatally [2]. Tissue stem and progenitor cells of each organ will be discussed in the later section on clinical application. Various strategies to engineer translatable organs utilising stem cell biology, including novel cell culture methodology, gene editing technology, tissue engineering and biomaterial technologies, will then be reviewed. It is beyond the scope of this review to discuss the entire body of research on biomaterials. Instead, we will describe the surgically-relevant part of these technologies of biomaterials in relation to stem cell biology and tissue engineering approaches, as biomaterials are now an indispensable part to tissue engineering. Next, relevant paediatric surgical preclinical and early studies aiming to alter underlying pathophysiology and to replace damaged organs will be reviewed by organ system. Finally, potential future outlooks in the field and major hurdles for clinical translation will be discussed.

## Overview of technological platforms in regenerative medicine

Regenerative medicine aimed to overcome three overarching themes: (1) to facilitate the repair of damaged tissues, (2) to replace damaged cells with healthy functional cells, and (3) to replace the damaged organ system with engineered tissues. There have been many technological advancements to this aim, including tissue engineering, stem cells and organoid biology, and gene editing. These advancements have allowed overcome significant technical hurdles and led to a more profound knowledge of how resident cells communicate with their microenvironment, issues of immunogenicity, and the role of stem or progenitor cells in tissue regeneration.

**Fig. 1:**
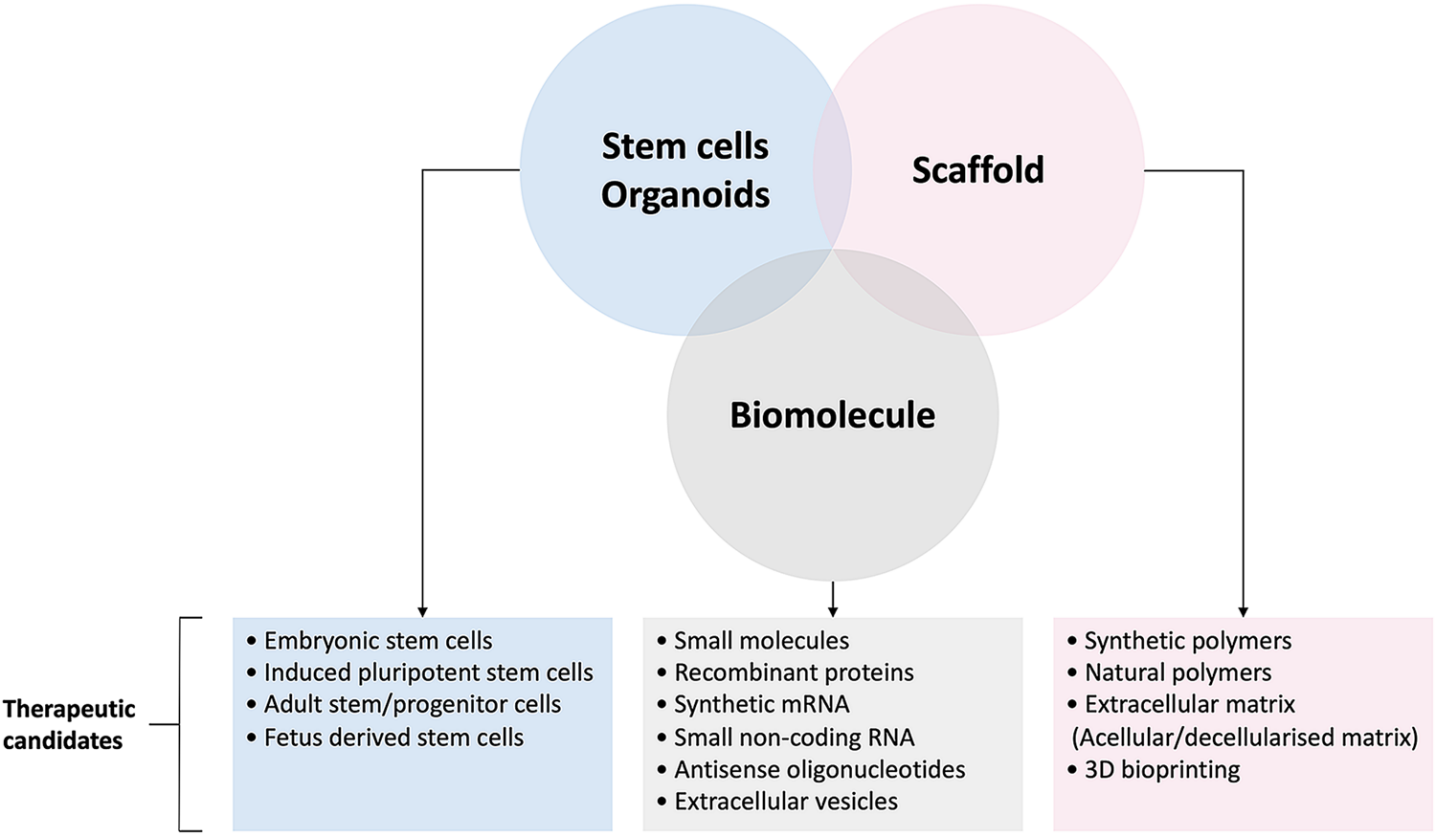
Schematic representation of different aspects of tissue engineering and therapeutic candidates

The main components of successful bioengineered tissues include cell sources, scaffolds, and biomolecules (Fig. 1). Although primary cells are ideal cell sources for regenerative medicine, they are usually in short supply, particularly in damaged organs, and do not proliferate enough to provide sufficient cell yield for clinical application. Stem cells can proliferate, differentiate, and have a self-renewal capacity, all of which are ideal for therapeutic application. Stem cells can be derived from native stem and progenitor populations, embryonic stem cells (ESC), or induced pluripotent stem cells (iPSC) from healthy or damaged tissues [3–6]. Furthermore, these stem cells can be genetically modified or corrected using gene editing technologies (such as the CRISPER-Cas system) [7]. The second component is the biomaterials, which are pivotal in providing an optimal microenvironment for cell growth and functional differentiation. These biomaterials include extracellular matrix (ECM) derived from decellularised tissue, natural polymers, and 3D bioprinting [8–10]. The final component is biomolecules, such as growth factors and morphogens. These molecules can be introduced as small molecules, recombinant proteins, synthetic mRNA, small non-coding RNA, and extracellular vesicles (e.g., exosomes) [11]. Some of these molecules can be incorporated into the scaffold while fabricating tissues, and the release can be controlled.

Recently, in vitro human organ models with physiological functions similar to native organs have become feasible through engineering research, such as micro-physiological systems represented by organ-on-chip. Among in vitro models, organoid-based platforms have a wide range of applications, from basic research in embryology and regenerative medicine to disease modeling and drug discovery research. Furthermore, the application of organoids to regenerative medicine using organoids for transplantation is also beginning to be explored.

**Fig. 2:**
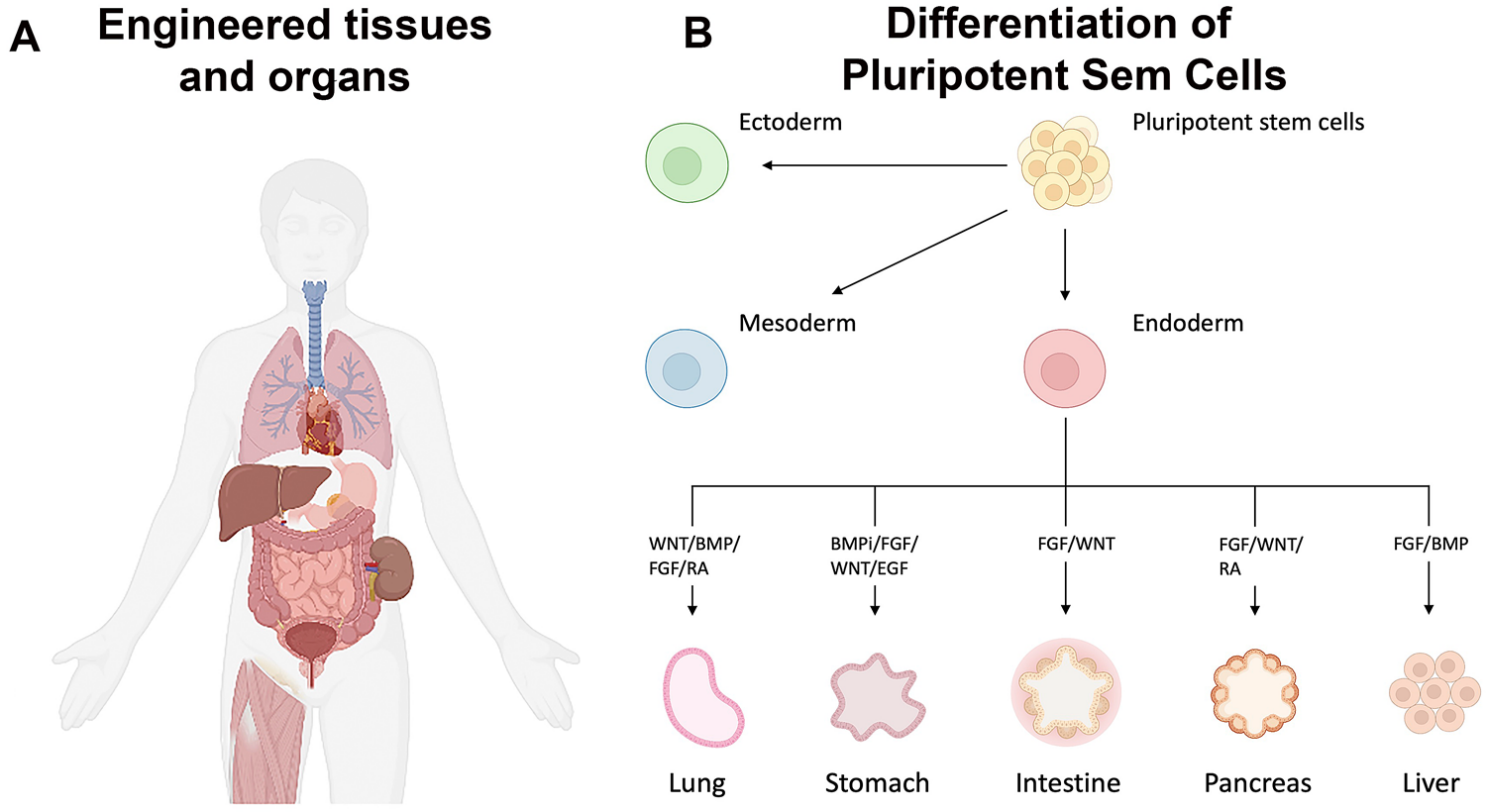
Regenerative medicine approach using somatic cells or pluripotent stem cells: schematic of various organs which can have been engineered **A** and organoids that can be grown from stem cells and the tissue-specific developmental signals that are employed **B** Adapted from Clevers, 2016 [12] and created with BioRender.com

Organoids have three-dimensional structural characteristics similar to those of organs in vivo. Since organoids are composed of multiple differentiated cell types, they are expected to have physiological responses similar to native tissues compared to the “classic” cell culture [12]. Organoids can primarily be derived from adult stem cells or pluripotent stem cells such as ESCs or PSCs [12]. By artificially reconstituting embryological processes in vitro, organoids of various organs of ectodermal, mesodermal, and endodermal lineages have been created (Fig. 2) [13–21].

An ultimate application of organoids in tissue engineering is the regeneration of deficient tissues through organoid transplantation. Improved survival of liver failure models through liver organoid transplantation and regeneration of intestinal epithelium through intestinal organoid transplantation have been reported [21–23]. Organoid transplantation is hoped to advance the treatments for critically ill paediatric patients with limited therapeutic options, such as biliary atresia and intestinal failure.

## Therapeutic application relevant to paediatric surgery

### Trachea and lung

Airway systems encounter two main fields of interests with substantial unmet clinical needs: conduit system and parenchymal disease. Severe forms of congenital tracheal stenosis/agenesis, laryngotracheal clefts, trauma and aggressive forms of cancer may require the replacement of main airways [24]. In paediatric population, cystic fibrosis and lung hypoplasia caused by bronchopulmonary dysplasia or congenital diaphragmatic hernia (CDH) are instead the leading causes of untreatable parenchymal disease. Allogenic transplantation is the ultimate treatment option for end-stage lung diseases, but it is still associated with a significant burden of morbidity and mortality and relies on donated organs [25].

#### Stem and progenitor cells

The human respiratory system is composed of a highly complex and hierarchical structure from proximal conducting region to distal alveolar region with numerous different types of cells along [26], varying according to the needs and functions each region fulfils. Epithelial cells in the airway are usually quiescent, but progenitor cells retain the potential to propagate and restore the epithelial integrity following injury [27]; these are classified as endogenous lung stem cells. Exogenous stem cell sources originating from other adult tissue or pluripotent stem cells may also be used for airways regeneration.

In early development the digestive and respiratory systems share a common precursor from the anterior foregut endoderm (marked by the expression of the transcription factor Nkx2.1). These progenitors are later specified by transcription factors Sox2 and Sox9 in the proximal and distal regions respectively. Despite the mesenchymal instructive paracrine signals are crucial to regulate proliferation and maturation of endodermal progenitors, there is a paucity of evidence concerning the airways mesodermal-derived progenitors, which are nevertheless thought to share a common origin with cardiopulmonary mesoderm progenitors [28].

In the mature proximal conducting region of the human lung, TRP63 + KRT5 + basal cells have been identified as a progenitor cell population [29]. However, to-date little is understood about the molecular mechanisms for basal cell self-renewal and differentiation, yet recent evidence in murine models indicated Notch signalling plays an important role [30]. Various protocols have been described for the isolation and culture of these human basal cells, with the most efficient methods being co-cultures of fibroblast with feeder layer and/or specific signalling pathway inhibitors [31–33].

In the alveolar region, alveolar type 2 (AT2) surfactant-producing cells are widely recognised as the best progenitor candidate [34–36]. In addition, several recent studies have suggested another stem/progenitor cell candidate in the mouse, such as Trp63 + Krt14 + basal cells or alveolar epithelial cells expressing the laminin receptor α6β4 [31, 37].

As exogenous stem cell sources promising candidates are bone marrow-derived stem cells [38], ESCs [39–41], human iPSCs through combination of FGF, BMP and Wnt signalling pathways [18, 42, 43] and human amniotic fluid stem cells (AFSCs) [44]. For instance, administration of hAFSCs into hypoplastic lungs in a nitrofen-induced CDH rat model rescued lung growth, bronchial motility and innervation [45].

#### Three-dimensional culture system

Most evidence on respiratory stem cell biology had been using 2D cell culture models, clearly too simplistic to reflect the complex in vivo interconnections and microarchitectures. Development of lung organoids has been reported from the different epithelial cell populations, including basal cells, club cells and AT2 cells, in addition to hPSCs [46]. A variety of names have been given to these organoids including tracheosperes, bronchospheres, alveospheres, alveolar spheroids, depending on the site of origin of the cells [46–49]. Recently, SFTPC + AT2 cell-like alveolar stem cells induced from hPSCs in organoids showed long-term stable expansion while maintaining their stem cell properties by utilising a co-culture system with fibroblasts [50] and smooth muscle cells [51].

Air–liquid interface (ALI) culture systems provide cultured cells with a more physiological environment and allow better differentiation for induced pluripotent stem cells [52]. Since the first model described in 1988, ALI has enabled the development of disease models such as asthma and cystic fibrosis [53, 54]. Further studies combining the advantages of 3D-organoid system with ALI culture may lead to the development of a more sophisticated in vitro miniature lung model.

#### Tissue engineering

##### Tracheal engineering

The upper airway has been thought to be relatively amenable to bioengineering due to its simple structure and function, and has already been engineered and translated clinically on a compassionate basis [55, 56]. Most successfully engineered upper airways involve co-culturing of different cell lines such as autologous bone marrow-derived mesenchymal stem cells (MSCs) and airway epithelial cells on a decellularised trachea scaffold [56, 57]. Alternatives for decellularised trachea include allogeneic trachea [58], aortic grafts [59] and composite reconstruction from autologous tissue.

##### Whole lung engineering

Whole lung engineering was obtained in rodent lungs by repopulating decellularised lung ECM with epithelial and endothelial cells and allowed gas exchange [60–62]. More recently, a scaling up in size was obtained successfully engineering human-sized lung by reseeding porcine decellularised lung with human airway epithelial progenitor cells and human umbilical vein endothelial cells in a bioreactor system and achieving a surgical implantation into porcine recipients [63]. An additional promising technique is bioprinting, the deposition of layers of different cell types and matrix material, addressing the need for creation of complicated tubular airflow and vascularity paths [64].

### Oesophagus

In paediatrics, many conditions may require the presence of a complete or partial substitute of the oesophagus, including long-gap oesophageal atresia (LGOA), caustic ingestion and traumatic injuries. Despite great advances in clinical care, reconstruction of oesophageal continuity is still associated with substantial morbidity and severe consequences on quality of life [65].

Research on tissue engineering of oesophagus was preceded by advancement of biomaterials, including decellularised, natural derived and synthetic materials. The successful in vivo implantation of these materials, using the small patch implantation model, has demonstrated their biocompatibility [66]. However, when aiming to generate longer oesophageal constructs, spontaneous migration of adjacent cells is not sufficient and identification of suitable oesophageal cell populations for bioengineering and establishment of their in vitro isolation and expansion method is an essential prerequisite for successful clinical translation [67]. Other obstacles for functional oesophageal replacement include the need for vascular supply, crucial for reseeded cells survival, and innervation of the implanted tissue, to enable efficient peristalsis of food intake.

#### Stem cells of oesophagus

##### Development of oesophagus from early foregut

While ventral foregut endoderm expresses transcription factor Nkx2.1 giving rise to the respiratory system, transcription factor Sox2 and Pdx1 are vital signalling pathway molecules for specification towards oesophagus, stomach and pancreas [68].

##### Stem and progenitor cells

*Epithelial cells*: The continuous turnover of the epithelial layer of the oesophagus relies on the so-called basal layer. These cells mature flattening into a stratified squamous epithelium consisting of 20–30 layers [69, 70]. Recent advances in lineage-tracing technology have revealed the stem/progenitor function of KRT5 positive basal cells in mice [71]. Currently, prevailing models for basal cell homeostasis in mature oesophagus are two: the heterogenous model, with multiple populations of basal cells (low integrin b1 and high laminin b2 [72] and CD34 + [73] subpopulations being the most validated), versus the homogenous model, with a single progenitor population maintaining self-renewal and differentiation capacity [74–76]. More recently, other various markers which indicate basal cell subpopulations have been advocated, although their overlapping has not been determined [77–79]. Further analysis to clarify relationships between these markers and the genuine indicators for stem cell populations is required. As an alternative approach, some researchers successfully used oral epithelial cells as an alternative cell source readily obtained during a minimally invasive procedure, with a high similarity in phenotype to oesophageal epithelial cells [80, 81].

*Muscle cells*: The submucosa and muscularis externa comprise mesenchymal elements of oesophageal wall; nerve fibres and ganglion cells are responsible for the generation of coordinated peristalsis [82, 83]. However, the origin of cells forming oesophageal muscular layer also remains an unanswered question. Initially, the unique structure of the human oesophageal muscle layer, a mixture of skeletal and smooth muscle, was considered to be the result of transdifferentiation of smooth muscle into skeletal muscle [84, 85]. However, lineage-tracing studies provided this assumption wrong [86]. Alternatively, it has been suggested that a skeletal muscle population originating from cranial mesoderm may migrate into the smooth muscle layers [87–89].

#### Tissue engineering

Much interest in tissue engineering of the oesophagus has focused on using acellular scaffolds derived from animal or human tissues and with or without cell-seeding. In particular, difficulty mimicking the complex native structure using synthetic materials whilst satisfying all the crucial features required for transplantable tissue constructs has provoked tissue engineering research to shift towards decellularised scaffolds [90, 91]. Badylak et al. demonstrated promising regenerative advantage of small intestinal submucosa (SIS) laid onto the surface of dissected submucosal layer following endoscopic submucosal resection in a human clinical trial [92]. In an attempt to generate a cell-based construct which more closely resembles the native structure of the oesophagus, researchers have attempted combination of synthetic and cells such as polyglycolic acid (PGA) and human amniotic membrane loaded with oral keratinocytes, fibroblasts, and smooth muscle cells in the canine model [93], acellular matrix recellularised with skeletal myoblasts covered by human amniotic membrane seeded with oral keratinocytes in a minipig model [94]. Urbani et al. developed recellularised oesophageal grafts using mesangioblasts and fibroblasts that formed a muscle layer after dynamic bioreactor culture [95–97]. Epithelial progenitor cells and enteric neural crest cells were functionally engrafted, and the graft was successfully prevascularised in vivo [95]. More recently, tubular synthetic scaffolds were seeded with autologous mesenchymal stem cells in preclinical models [98] and clinical application [99].

### Stomach

Gastrectomy is commonly performed in adults and children for a variety of conditions, ranging from the need of an oesophageal substitute as in LGOA or caustic ingestion [100] till gastric cancer [101]. After gastrectomy, patients suffer from various symptoms due to a loss of reservoir, digestive and exocrine glandular functions [102]. To compensate for the loss of reservoir, various techniques have been developed but the lack of gastric mucosa, which plays a crucial role for the functionality of stomach, remains an unsolved aspect [103].

#### Stem cells and progenitors

*Epithelial cells*: Organogenesis of the stomach originates from a highly coordinated interaction between foregut endoderm and splanchnic mesoderm due to interplay of various factors and signals, such as HHEX, SOX2, retinoic acid, FGF and Wnt pathways and gradients of BMP [104].

The adult stomach is lined with a glandular epithelium in which self-renewal is driven by gastric stem cells. However, their exact location among the glandular functional unit is not clear. Using incorporation of labelled nucleotides in animal models, historical studies suggested that renewal of antral gastric cells was driven by one or a few cells localised in the isthmus [105]. More recently, by adapting lineage-tracing research from the previous intestinal studies, Clevers et al. identified a small number of cells at the bottom of pyloric gastric niches with active Wnt signalling marked with Leucine-rich repeat-containing G-protein coupled receptor 5 (Lgr5) [106, 107]. These cells were able to form and maintain fully differentiated gastric organoids. Subsequently, Sigal et al. identified the presence of a second stem cells population, by targeting the classic Wnt gene Axin2 [108].

When combined with Lgr5 labelling, AXIN2 + LGR5 + and AXIN2 + LGR5- subpopulations located at the base and lower isthmus of the gastric glands were identified. Importantly, the two cell types can replenish each other, being alternatively activated and silenced in case of specific depletion [109]. Moreover, among all Wnt signalling, R-spondin 3 secreted by a-SMA positive mesenchymal cells beneath the gland has been pinpointed as an essential guide to epithelial stem cell renewal, in a reciprocal interaction [108].

Finally, in 2014 McCracken et al. firstly described the de novo generation of gastric mucosa from human iPSCs by temporal manipulation of the abovementioned signalling pathway [19] and subsequently obtained to recreate a functional epithelium [110].

*Smooth muscle cells*: The smooth muscle of the stomach is thicker than that of other digestive organs, but the mechanisms of stomach-specific myogenesis from a pool of progenitors are not fully understood [111]. Moreover, enteric neural crest cells also have a role in stomach smooth muscle development [112]. For example, specialised muscle cells in the pyloric sphincter integrate neuronal and hormonal signals to control the progression of food into the intestine [113].

#### Tissue engineering

Tissue engineering of the stomach for translational medicine has two main aims: regeneration of epithelial lineage itself and physiological reinforcement of gastric wall [114]. In the literature, different approaches have so far been applied: historically, scaffold biomaterials, such as collagen sponges or nonwoven poly glycolic acid (PGA) fibres, with or without cells loaded on the inner surface have been commonly used [115]. However, the regeneration achieved was just partial with a disturbed epithelialisation, a non-functional muscular layer and a significant contraction over time [116]. Recently, Tanaka et al. demonstrated that a myoblast cell sheet without scaffold could prevent leakage of the enteral contents in the mice model and obtained a rapid recovery of the discontinuation [117].

In addition to the reinforcement of the gastric wall defect, improvements in the functional regeneration of the mucosa have been seen once the scaffold has been loaded with organoids. Vacanti's group seeded organoids of a mesenchymal cores surrounded by epithelia onto a PGA tubular construct and then implanted both small (2-step replacement of native stomach in the murine model [118]) and large animal models (in the porcine model the constructs were implanted intraperitoneally [119]). To our knowledge, present literature is lacking naturally derived scaffolds, which may potentially facilitate regeneration, as composition of ECM in each tissue is unique and it could influence cell migration and differentiation.

### Intestine

Various paediatric gastrointestinal diseases, including necrotising enterocolitis, gastroschisis, midgut malrotation and volvulus, intestinal atresia, and inflammatory bowel diseases, may result in short bowel syndrome (SBS), in which limited length of the intestine cannot perform the required functions such as absorption and barrier function, commonly caused [120]. Patients with SBS eventually develop intestinal failure when the residual bowel can no longer have sufficient absorptive capacity to meet their nutritional needs and become dependent on total parenteral nutrition associated with poor quality of life, risk of sepsis and liver dysfunction, and mortality [121]. Although small bowel transplantation is the ultimate cure and has already been performed in more than 2000 cases, surgical outcomes still remain unsatisfactory, with the survival rate at five years around 50% [122].

#### Intestinal stem cells and progenitor cells

The intestine is a highly complex organ with several essential functions; nutrient absorption, host immunity, and mucin secretion [123]. The intestinal epithelium has an integrated crypt-villus structure comprised of different cell types with specialised functions. The characteristic high turnover rate of the intestinal epithelium is mediated by intestinal stem cells located in the crypt base [124]. The aligned musculature and innervation are crucial to achieving synchronised and coordinated contraction throughout the enormous gastrointestinal tract. Smooth muscle cells and the enteric nervous system, together with the interstitial cells of Cajal (ICCs) as pace-making cells, mediate this function [125]. The vascular and lymphatic systems play a role in both absorption of nutrients and the maintenance of vascular supply [126].

Organogenesis and maturation of the intestine were recently reviewed in [127], emphasising the role of signalling pathways on intestinal specification, gut tube patterning, integration of smooth muscle and enteric nervous system (ENS), crypt-villus formation and postnatal maturation and maintenance. For example, the foregut and hindgut are specified by the expression of Sox2 and Cdx2 [128, 129]. Wnt and Notch signalling are two major signalling pathways regulating intestinal stem cells [130, 131]. This knowledge of molecular pathways, such as Wnt and Notch signalling, has been adapted into successful epithelial cell culture protocols.

##### Stem and progenitor cells

Generating functional intestinal epithelium is crucial to restoring a patient's enteral autonomy. Barker et al. first identified a widely accepted intestinal stem cell marker, Leucine-rich repeat-containing G-protein coupled receptor 5 (Lgr5), expressed in crypt base columnar cells, and demonstrated that a single Lgr5 + cell has the capacity to regenerate crypt-villus structures [124, 132]. Mesenchymal-free 3D culture systems allow intestinal stem cells to form enteroids or organoids with intestine-specific crypt-villus architecture by combining niche factors [132]. These mouse organoids were successfully repopulated onto a damaged colonic epithelial layer in vivo when delivered via a simple colonic enema, indicating their potential for clinical application for intestinal regeneration [23]. Pluripotent stem cells are alternative cell sources for regenerating human intestinal epithelium. Human iPSCs were successfully differentiated into definitive endodermal cells, followed by hindgut specification [20]. When transplanted under the mouse kidney capsule, enhancing vascularisation, these cells formed mature organoids with terminally differentiated intestinal cell lineages and adult-type crypt-villus architecture [133].

The mesenchymal and smooth muscle components of the intestine represent another challenge for intestinal regeneration, as they have a crucial role in maintaining the epithelial niche. Recently, human iPSC-derived intestinal organoids, in combination with human-PSC-derived neural crest cells, were reported to generate a smooth muscle layer that is positive for the smooth muscle marker desmin and can functionally respond to electrical and pharmacological stimuli [134].

Large-scale vascularisation capable of enabling successful surgical anastomosis into isolated engineered intestinal segments remains a challenge. Kitano et al. successfully repopulated human endothelial cells and human iPSCs-derived intestinal progenitors on a decellularised intestinal scaffold in a rat model. They restored perfusability and patency following in vivo isolated transplantation, as well as in ex vivo perfusion testing [135].

#### Tissue engineering

To create a sizable intestinal equivalent that can be successfully anastomosed to the native intestine, tissue engineering of the intestine has attempted to bring some or all cellular components and systems together into one platform. Given the complex structure and highly organised function of the intestine, the creation of synthetic biomaterials which recapitulate intestinal ultrastructure, such as the crypt-villus structure and stem cell niche, is challenging. Decellularised scaffolds of the intestine have attracted attention with advantages over synthetic materials. Our group has established a detergent-based decellularisation protocol that retains essential ECM proteins, angiogenic properties, and mechanical strength [90]. Recent studies focus on the integration of intestinal organoids into tissue-engineered intestines. Both human and mouse organoids replicated the structure and absorptive function of the intestine when implanted in a mouse model [136]. Implantation of human intestinal organoid-seeded scaffolds results in a tissue-engineered intestine resembling a native structure [137, 138].

### The enteric nervous system (ENS)

Neurological dysfunction within the enteric nervous system (ENS) is responsible for a wide range of gastrointestinal motility disorders, such as oesophageal atresia [139], oesophageal achalasia [140, 141], and most commonly, Hirschsprung's disease (HD) and intestinal aganglionosis in children [142, 143]. Specifically, HD is known to be the result of impaired migration of enteric neural crest cells (NCCs) during intestinal development, with the primary symptom being intestinal obstruction caused by the lack of peristalsis [144]. Surgical resection of the aganglionic segment is the standard therapy; however, it is often associated with persistent symptoms, such as repeated enterocolitis and dysmotility [145, 146]. The restoration of the ENS through stem cell transplantation is attracting attention as a potentially curative therapeutic method [147].

Recently, substantial progress has been made in ENS research through the identification and isolation of enteric neural stem cells (ENSCs): multipotent cells which can be isolated from human GI tracts. McCann et al. reported the restoration of the ENS and colonic motility following the implantation of ENSCs into a mouse model of human enteric neuropathy (neuronal nitric oxide synthase deficient mouse), which highlighted the significant possibility of clinical ENSC therapy [148]. PSCs are alternative sources to circumvent the above issue, with abundant proliferative capacity. Fattahi et al. successfully isolated ENS progenitors from human PSCs and demonstrated their maturation into functional ENS [149]. They also reported restoring ENS components on human PSC-derived intestinal organoids by combining human PSC-derived NCCs in 3D growth conditions. Co-implanted NCCs migrated into intestinal mesenchyme and functionally integrated into the intestinal smooth muscle of the intestinal organoids [134, 150].

### Pancreas

The intense interest in understanding human pancreas development is related to the treatment of diabetes, a growing health problem worldwide. Type 2 diabetes, the most prevalent form of this disorder, is typically characterised by a progressive failure of β-cells to meet the body’s demands for insulin; in contrast, β-cells are lost by autoimmune destruction in type 1 diabetes mellitus. Both mechanisms leading to diabetes are related to genome sequence variants in adult β-cells [151] and during developmental events [152]. In both cases, cell therapy and tissue engineering of the whole or partial organ are of great interest for potential transplantation or drug development.

#### Development of pancreas

Human pancreas formation starts with dorsal bud formation deriving from endoderm, and it continues with the appearance of the ventral bud. After the posterior migration, the ventral bud fuses with the dorsal pancreatic bud, giving rise to most of the pancreas. Although knowledge of events in human pancreas organogenesis is limited, Jennings et al. recently reported that contact between the dorsal pancreatic endoderm and the notochord leads to the exclusion of sonic hedgehog (SHH) expression from this endodermal region [153]. As a result, expression of the pancreatic and duodenal homeobox 1 (PDX) gene, a master gene for pancreatic development, is upregulated. Although all of the downstream effectors of pancreas development have not been determined yet, it appears that expression of the specific homeobox genes of the PAX family guides differentiation towards endocrine cell lineages that are known as α (glucagon), β (insulin), δ (somatostatin) and γ (pancreatic polypeptide) cells. By contrast, little is known about exocrine pancreas development [154].

#### Stem cells in the pancreas

Pancreatic progenitor cells arise from a cluster of cells originating from the developing foregut. These clusters have been characterised to show multipotent properties [155] and the ability to differentiate into endocrine, exocrine, and ductal lineage precursors [156]. More recently, adult pancreatic stem cells have been found as a subpopulation of biliary tree stem cells, precursors of both hepatic and pancreatic stem cells in the hepato-pancreatic common duct [157, 158]. MSCs can differentiate into immature islet-like cells with such low efficiency that they are not a practical source for clinical products [159].

#### Tissue engineering

Since the first experiments pursuing pancreatic differentiation [160], there has been a number of differentiation protocols using hESCs [161] or hiPSCs [162, 163], focusing on the generation of mature, single hormone-expressing, glucose-responsive human β-cells [164]. However, some transcriptional similarities have been identified between PSC-derived β-cells and human foetal β-cells, which may explain a relative immaturity of PSC-derived β-cells [165]. This immaturity may also reflect the lack of an appropriate developmental niche containing the necessary signalling factors for pancreatic cell differentiation [164], consistent with the better maturity achieved by in vivo incubation in mouse [157, 161] or culture on synthetic scaffolds [166]. In this context, some preliminary results are highlighting niche-derived influence on cell differentiation [167], although the whole mechanism remains to be clarified [168]. Some latest protocols have significantly improved in generating a higher proportion of monohormonal cells [169]. However, it is unclear whether these cells possess the ability of glucose-responsive insulin secretion and this remains a vital requirement for clinical transplantation [170].

### Diaphragm and skeletal muscle

Several different approaches have been attempted to repair large defect in congenital diaphragmatic hernia (CDH), including synthetic mesh materials, autologous thoracic or abdominal muscle flap, with PTFE/Gore-tex^®^ being the current standard practice. Because synthetic materials are related to various long-term complications, recent studies described using naturally derived materials, such as small intestine submucosa (SIS) and acellular dermis, with a failure of regeneration and a resultant re-herniation [171, 172]. A new strategy for a more effective biological substitute applicable for diaphragmatic defect repair or other muscular-related diseases (such as abdominal wall defect, trauma, volumetric muscle loss, or genetic disorders) represents a significant challenge.

#### Skeletal muscle regeneration

Skeletal muscle retains high regenerative capacity upon mechanical damage or diseases. This regenerative ability is mediated by a resident stem cell population for skeletal muscle, named muscle satellite cell (SC), and identified by the transcription factor Pax7 [173, 174]. The repair and regeneration of skeletal muscle comprise three phases: (1) host inflammatory cells are recruited to a damaged site in the inflammatory phase. (2) In the repair phase, pro-inflammatory macrophage (M1) is gradually replaced by anti-inflammatory macrophage (M2), which contributes to the proliferation and differentiation of SCs and their progeny; myoblasts [175]. (3) In the remodelling phase, newly formed muscle fibres are joined into the existing muscles [176].

#### Stem cells for skeletal muscle therapy and bioengineering

SCs and myoblasts are physiological skeletal muscle precursors, so it is rational that most studies on muscle cell therapy have been performed on these cell populations. Although investigations on SCs have demonstrated their proliferation and multipotent differentiation capabilities, utilising SCs for human cell therapy and bioengineered skeletal muscle has faced severe challenges and limitations [177]. Early-phase clinical studies showed that insufficient cells could be obtained from human biopsies, poor expansion potential of SCs cultured in vitro, poor survival in vivo, and low contribution of implanted SCs on muscle regeneration [178, 179].

Notably, in the setting of congenital disorders, SCs and myoblasts are exhausted, hindering efficient cell isolation and expansion in vitro. These drawbacks of SCs and myoblasts have led to the identification of alternative stem cell candidates for skeletal muscle regeneration, including mesangioblasts/pericytes [180], CD133-positive cells [181], Aldehyde dehydrogenase 1A1 (ALDH)-positive cells [182], MuStem cells [183] and hPSC [184].

Regarding the microenvironment of stem cell niche, the importance of mechanical rigidity of substrate used during in vitro culture has been noted to preserve satellite cell capacity. Appropriate elasticity or geometric micropatterning of a substrate where cells cultured on enhanced survival, self-renewal, and engraftment abilities of satellite cells in vivo [185, 186], proposing the requirement of investigation on optimal bioengineered stem cell niche [187].

#### Tissue engineering for skeletal muscle regeneration

In the setting of congenital muscular defects such as CDH or abdominal wall defects, a purely cell-based (scaffold-free) strategy is not applicable due to the massive skeletal muscle defects seen in many cases, which overwhelms the inherent regenerative potential. A bioengineered construct comprising exogenous myogenic stem/progenitor cells combined with scaffold biomaterials would be an attractive option for reconstructing these large defects. Growing evidence has suggested appropriate biomaterials can enhance the potential of stem/progenitor cells (see comprehensive review in [188]).

Most of these biomaterials are broadly divided into synthetic and naturally derived materials. Among various synthetic biodegradable materials, polycaprolactone (PCL), PGA, and poly-lactide-co-glycolic acid (PLGA) have been the most frequently tested materials in vitro and in vivo animal model, with relatively disappointing outcomes due to a pro-inflammatory response of the host immune system against these materials [189–192]. In contrast, an acellular ECM scaffold obtained by decellularisation of native muscle tissues is advantageous in mimicking native extracellular environments preferable for precursor cells, such as mechanical strength, microarchitecture, and growth factors.

##### Two approaches

Researchers have investigated an acellular approach, where only decellularised scaffold was used without cells. Sicari and Badylak et al. utilised an acellular scaffold derived from a porcine small intestinal submucosa-ECM and urinary bladder matrix for rodent volumetric muscle loss (VML) model and human patients. They showed constructive regeneration of skeletal muscle tissue [193, 194]. Piccoli et al. performed orthotopic transplantation of an acellular ECM scaffold derived from diaphragmatic muscle and demonstrated improvement of diaphragmatic function in an atrophic mouse model [195]. Based on results from preclinical trials, the first human clinical study implanting acellular ECM into severe VML cases have conducted, demonstrating early functional improvements and site-appropriate remodelling [194, 196].

In contrast, a cell-based tissue construct combining stem/progenitor cells and acellular ECM could be a better therapeutic solution, especially when the recipient's muscle is severely compromised and a scarcity of muscle progenitor pool [197, 198]. The combination of bone marrow-derived MSCs and acellular ECM demonstrated increased regeneration of muscle fibres and blood vessels [199].

## Future perspectives

In the last decade, regenerative medicine has expanded dramatically, combining numerous relevant fields and technologies, including stem cell biology, biomaterial engineering, immunology, and genetics, indicating the interdisciplinary nature of this emerging discipline. Many novel therapeutic strategies have been proposed to treat adult and paediatric pathologies and realise finely tuned transplantable "artificial" organs. However, regarding applying this innovative paradigm to the paediatric surgical clinical practice, significant practical and ethical limitations still need to be addressed, including optimisation, scalability, and manufacturing standardisation [200].

Recent breakthroughs in this field might provide various alternative platforms and therapeutic options. Since 2017, researchers have developed an artificial placenta system comprising an extracorporeal circuit, oxygenator, and fluid-filled womb-like environment, and they proved the concept using premature animal models [201, 202]. Another promising alternative is xenotransplantation of genetically modified organs. In January 2022, the first pig-to-human heart transplantation was conducted at University of Maryland School of Medicine and the University of Maryland Medical Center. The transplanted xenogeneic heart was obtained from genetically modified pig [203]. Although there are still significant obstacles to overcome before the eventual clinical application of these platforms, they could facilitate the development of novel therapeutic approaches or a deeper understanding of the underlying mechanism in regenerative medicine.

## Conclusion

The last decade has witnessed remarkable advance in both the basic knowledge and technologies in stem cell biology and bioengineering, which has made it possible to create a variety of engineered tissues and organs with the clinical translational potential. Due to the complexity of reconstruction in paediatrics, many paediatric surgeons contributed to the development of the field. In 1954 Dr Joe Murray, a paediatric plastic surgeon from Children's Hospital Boston, who subsequently received the Nobel prize in 1990, and his team, performed the first successful kidney transplant where the donor and the recipient were two identical twins [204]. Dr Judah Folkman, the father of angiogenesis research, was the director of the Vascular Biology Program and a former surgeon-in-chief at the Children's Hospital Boston. He had the ability to bring together both a scientist's and a surgeon's perspectives to finding solutions to medical problems. Dr Folkman published his ideas about angiogenesis in 1971 [205]. Working together in his laboratory at that time were Dr Jay Vacanti and Dr Robert Langer who started collaborating to create new matrices and scaffolds that could allow cells to be expanded and to differentiate. They defined the concept of tissue engineering as a new field that applies the principles of biology and engineering to the development of functional substitutes for damaged tissue [9]. Dr Folkman’s mentorship influenced many working in Boston at that time and just a few floors below in the same building Dr Anthony Atala, engineered the first human bladder which was later implanted in children requiring bladder augmentation [206]. Dr Prem Puri contributed as well to the field by investigating many of the diseases which will ultimately benefitting of a regenerative medicine approach like Hirschsprung’s Disease, congenital diaphragmatic hernia and abdominal wall defect. Moreover, we can identify Prof Puri as one of the contributors to the field by introducing and popularised the correction of vesicoureteric reflux by endoscopic injection of polytef paste. The bulging mechanism created by the polymer can be considered a first step towards an engineering approach which is safe, simple, and effective in correcting all grades of reflux [207].

Despite the enormous progress, the field of regenerative medicine is still in its relative infancy phase: however, with present momentum on research progress and interdisciplinary collaboration, it is reasonable to expect that shortly, regenerative medicine will lead to realising unprecedented surgical therapeutic strategies where impaired organs are replaced by novel biological substitutes.

## Data Availability

Due to the nature of the paper, no original data are presented.
